# Decision Trees for Predicting the Physiological Responses of Rabbits

**DOI:** 10.3390/ani9110994

**Published:** 2019-11-18

**Authors:** Patrícia Ferreira Ponciano Ferraz, Yamid Fabián Hernández-Julio, Gabriel Araújo e Silva Ferraz, Raquel Silva de Moura, Giuseppe Rossi, Jairo Alexander Osorio Saraz, Matteo Barbari

**Affiliations:** 1 Department of Agricultural Engineering, Federal University of Lavras (UFLA), Lavras, Minas Gerais 37200-900, Brazil; gabriel.ferraz@ufla.br; 2 Faculty of Economics, Administrative and Accounting Sciences, Universidad del Sinú Elías Bechara Zainúm, Montería, Córdoba 230001, Colombia; yamidhernandezj@unisinu.edu.co; 3 Department of Animal Science, Federal University of Lavras (UFLA), Lavras, Minas Gerais 37200-900, Brazil; raquelmoura@ufla.br; 4 Department of Agriculture, Food, Environment and Forestry, University of Firenze, 50145 Firenze, Italy; giuseppe.rossi@unifi.it (G.R.); matteo.barbari@unifi.it (M.B.); 5 Departamento de Ingeniería Agrícola Alimentos, Sede Medellin, Facultad de Ciencias Agrarias, Universidad Nacional de Colombia, Medellín 050004, Colombia; aosorio@unal.edu.co

**Keywords:** ear temperature, respiratory rate, thermal environment, rabbit breeder, welfare

## Abstract

**Simple Summary:**

The primary aim of this paper is to develop decision trees to predict rabbits’ physiological responses, such as the respiratory rate or ear temperature, based on environmental variables (dry bulb temperature and relative humidity). The decision tree for ear temperature exhibited better statistical indices, indicating the benefits of using the ear temperature as an indicator of thermal stress. Our findings confirm that the resulting decision trees are powerful classifiers, and the results can be easily understood. Hence, the proposed decisions trees can aid in investigating the influence of environmental conditions on physiological responses and, consequently, the rabbits’ welfare. These results can be used in practical situations and can be obtained in real time to support rabbit breeders in decision-making to improve environmental conditions for rabbits.

**Abstract:**

The thermal environment inside a rabbit house affects the physiological responses and consequently the production of the animals. Thus, models are needed to assist rabbit producers in decision-making to maintain the production environment within the zone of thermoneutrality for the animals. The aim of this paper is to develop decision trees to predict the physiological responses of rabbits based on environmental variables. The experiment was performed in a rabbit house with 26 rabbits at eight weeks of age. The experimental database is composed of 546 observed data points. Sixty decision tree models for the prediction of respiratory rate (RR, mov.min^−1^) and ear temperature (ET, °C) of rabbits exposed to different combinations of dry bulb temperature (t_db_, °C) and relative humidity (RH, %) were developed. The ET model exhibited better statistical indices than the RR model. The developed decision trees can be used in practical situations to provide a rapid evaluation of rabbit welfare conditions based on environmental variables and physiological responses. This information can be obtained in real time and may help rabbit breeders in decision-making to provide satisfactory environmental conditions for rabbits.

## 1. Introduction

The profitability of a rabbit farming system must consider the rabbit’s breed, nutrition, management, and sanitation as well as the thermal environment of the animal during the productive period [1]. Currently, increasing pressure has emerged from public opinion and consumers for farms to improve housing conditions and comply with animal welfare standards [2]. In addition, intensive housing systems have a direct influence on the comfort and welfare of the animals, due to the difficulty in maintaining a thermal balance inside the facilities and in allowing natural behaviours of the animals, affecting the productive performance [3].

For effective rabbit meat production, a thermoneutral zone (TNZ) of 15–25 °C should be maintained [4], with a relative humidity (RH) of 60–70% [5]. In regions with tropical and subtropical climates, joint problems arise from high indoor temperatures associated with a high RH of the rabbit house during the summer under imperfect ventilation conditions. Rabbits are much more tolerant of low temperatures than of high temperatures [6]. When subjected to severe heat stress and heat load increases, rabbits try to sustain homeothermy by using internal physiological means to re-establish a thermal balance [7]. According to [8], the respiratory rate (RR, %) and ear temperature (ET, °C) reflect the primary physiological mechanism for heat dissipation because most of the sweat glands in rabbits are non-functional and little perspiration (evacuation of water through the skin) occurs because of their fur [7].

The ET plays a vital role in the thermoregulation of rabbits [7]. The rabbit’s ear exerts a physiological function due to a vasomotor mechanism that controls blood circulation from the body core to the blood vessels and blood capillaries, which can be dilated and constricted by a vasomotor tool [9].

When the body temperature of the animal increases, it may cause acceleration in the breathing, attempting to dissipate the excess heat and maintain homeothermy. According to [10], when behavioural adjustments do not achieve a maintenance of homeothermy, animals exhibit physiological adaptations, with a change in RR being one of the first symptoms presented.

Therefore, the environment inside a rabbit house exerts a strong influence on the rabbit production system because it affects the physiological responses of the animals; thus, it is crucial to characterize the optimal environmental conditions for raising rabbits. Investigating the influence of the environment on rabbit physiological responses is vital to understand the effects of thermal conditions on rabbit breeding [10]. For these reasons, tools (models) are needed to assist rabbit producers in making decisions to maintain the production environment within the TNZ for the animals, in order to obtain maximum production.

Decision trees are one of the most potent and popular classifiers available, and because they follow a flow system similar to that of human logic and reasoning, decision trees can be trained [11,12,13,14]. According to [15], the accuracy of a decision tree is comparable to or higher than the accuracy of other classification models [16] because decision trees do not require a high number of parameters to be adjusted in their design [17]. Due to their intuitively appealing topology, the resulting classification or regression models are easy to comprehend [18,19].

Decision trees have been used as reliable techniques for the development of predictive models to support decision-making because they are hierarchical graphical structures that can be easily understood and applied. Decision trees are characterized by segmenting heterogeneous data according to their similarities such that the data become more homogeneous about the target variable [20,21].

For the above-mentioned reasons, the primary aim of this paper is to develop decision trees for predicting the physiological responses of rabbits based on environmental variables.

## 2. Materials and Methods

### 2.1. Housing and Animals

This experiment was conducted in the Sector of Rabbit Breeding of the Animal Science Department, Federal University of Lavras (Lavras, Minas Gerais, Brazil). In the trials, 13 female and 13 male hybrid New Zealand White × Botucatu rabbits at eight weeks of age with a body weight of 1310 (±290) g were used. This age is considered as the growing period between weaning and slaughter [22]. During this period, it is expected that the rabbits will gain weight.

The committee of animal research and ethics of the Federal University of Lavras (UFLA) approved all procedures used in this experiment (protocol number 026/2016).

**Figure 1:**
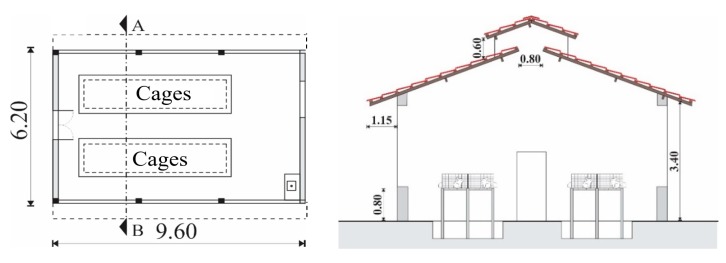
Rabbit house scheme. (**Left**) Floor plan of the rabbit house. (**Right**) Cut AB of the rabbit house section (B). Source: adapted from [1].

The rabbit house has the following measurements: 6.20 m wide, 9.60 m long, 3.40 m ceiling height, 0.80 m wall height, 1.15 m eaves. The roof is composed of clay tiles and has an inclination of 30° (Figure 1). The rabbit house is oriented along the directions of magnetic East–West. The house has a concrete floor and two cemented drainage ditches with dimensions of 1.50 × 6.00 m and a depth of 0.80 m.

The rabbits were housed in single cages (0.060 × 0.080 × 0.045 m). Water and a commercial feed with a constant formulation were provided ad libitum to the rabbits throughout the experimental period. The feed satisfied the nutritional requirements of the animals, as recommended by [23].

### 2.2. Environmental and Physiological Response Measurements

To characterize the thermal environment inside the house, the dry bulb temperature (t_db_, °C) and RH (%) at a height of 1.0 m inside the house were measured.

The environmental variables (t_db_ and RH) were evaluated using a digital thermal hygrometer (INSTRUTEMP^®^, mod. ITWH-1280, precision of ±0.1 °C and 1.0%). The sensors were positioned to form a 1.0 × 1.0 m grid, with a total of 48 data points inside the house.

The ET was measured at three different points in the animal ear (base, middle, and tip), and the average of the values was calculated. An infrared thermometer (Raytek Raynger ST, Raytek Corporation, precision of ±0.1 °C) was used, with the emissivity control set to 0.95. The RR was obtained by counting the movements of the flank of each animal for 15 s; this number was then multiplied by four to determine the number of breaths per minute. The RR was evaluated with the aid of a digital timer (±0.01 s). The environmental variables (t_db_ and RH) and physiological responses (ET and RR) of all rabbits were assessed three times a day for seven days during the periods P1 from 7:00 to 8:00 a.m., P2 from 12:00 to 1:00 p.m. and P3 from 5:00 to 6:00 p.m. These periods were chosen to assess different thermal conditions during the day.

### 2.3. Dataset

A database containing the raw data for t_db_ (°C) and RH (%) as input variables and the RR (mov.min^−1^) and ET (°C) as output variables was generated for the rabbits. The experimental database was composed of 546 observed data points obtained from the experiment.

#### Data Partition Method for Validation of the Models

For analysis, the complete dataset (100%) was randomly distributed among two subsets using random sampling as data partition method: The percentage for the first subset (training) was 70%, and for the second subset (validation) was 30%, following the methodology proposed by [24]. These parameters can be customized by the user to train the decision trees. Additionally, a further subset was used to test the overall system performance; this subset consisted of the mean values of the experimental measurements, according to the methodology proposed by [25]. In total, 21 mean values were used to test the decision tree performance (Table 1).

### 2.4. Data Preparation

The objective is to arrange the input and output variables in matrix form (*m* × *n*), where *m* represents the number of instances and *n* represents the number of variables [26]. Table 1 shows the mean values described in the previous section. The first two columns represent the input variables, and the third and fourth columns represent the output variables. A similar table was constructed with the complete dataset (546 data pairs) to apply the random data sampling method.

### 2.5. Decision Tree Model Development

Sixty decision tree models were developed to predict the RR and ET of rabbits exposed to thermal challenges of different intensities and durations. Ten repetitions for each type of predictor selection (“allsplits”, “curvature”, “interaction-curvature”) for each output variable were applied in the training process, indicating that each output variable has thirty decision tree models containing t_db_ and RH as input variables and RR or ET as the output variable. As mentioned above, the mean data values were used to assess the performance of the decision tree models. From these models, the model with the best performance was selected to derive the performance results. To develop the decision tree models, the MATLAB^®^ 2017a [27] software tool was used.

#### Decision Tree Model Parameters

From the selected decision tree models, the ET model has the settings reported in Table 2.

The RR model has the parameters reported in Table 3.

## 3. Results and Discussion

The parameters listed in Table 2 and Table 3 were automatically selected by the software tool. Descriptive statistical indices, such as the absolute and standard deviation, percentage error, coefficient of determination R^2^, standard error and root mean square error (RMSE), and histograms were computed to evaluate the effectiveness of the decision trees in predicting the physiological responses. The performance results of the decision trees for the test subset (mean dataset) are listed in Table 4.

**Figure 2:**
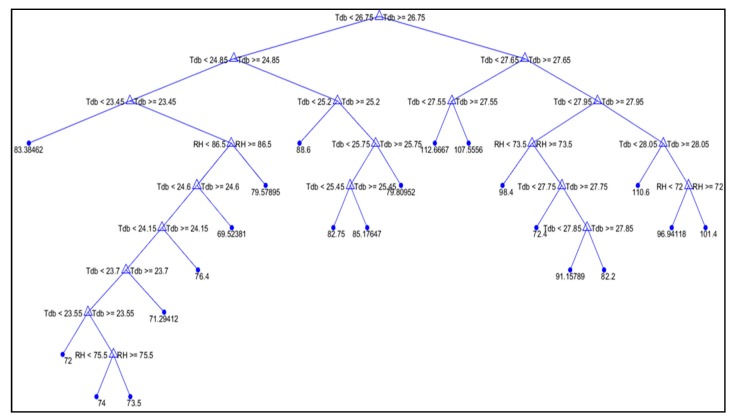
Visual decision tree for the RR output variable (mov.min^−1^).

**Figure 3:**
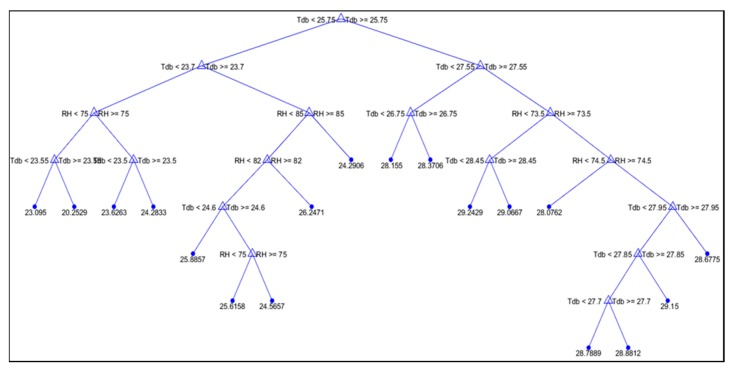
Visual decision tree for the ET output variable (°C).

The average structures of the RR and ET decision trees are depicted in Figure 2 and Figure 3, respectively. In both decision trees, two splits can be observed; the first split is related to t_db_ and the second split is related to RH, as the most important factors influencing the studied physiologic variables (RR and ET). According to the experimental data, these decision trees are related to t_db_ values of 23.4–29.2 °C and RH values of 67–87%, which represent common conditions in hot climates. In both decision trees, the first split for t_db_ occurs at approximately 25 °C, which is in accordance with reports affirming that the TNZ for rabbits is below 25 °C [4]. Conditions of stress induce behavioural and physiological modifications to enable rabbits to cope with the stressor [28].

According to the literature, the best parameter to assess for eventual stress conditions in rabbits is the ET, due to the vasoconstriction process that occurs in this part of the body [28,29]. The rabbit ear is the primary cutaneous vascular bed and plays a major role in temperature regulation [30]. According to [31], if t_db_ is low (below 10 °C), the animal will curl up to minimize the total area losing heat and to lower the ET. If the temperature is high (above 25–30 °C), the animal will stretch out to lose as much heat as possible by radiation and convection and to increase the ET. Additionally, when t_db_ is higher than the TNZ, the animal also pants to increase heat loss through water evaporation (latent heat). Sweat glands are not functional in rabbits, and the only controlled means of latent heat evacuation is achieved by altering the RR [31]. The RR is widely used and is considered the simplest variable for evaluating the physiological conditions of animals [32]; moreover, it has the advantage of being measured through visual analysis. The RR is an important parameter for evaluating an animal’s comfort and well-being because as t_db_ increases, the RR also increases to enhance heat dissipation [33].

Based on Figure 2 and Figure 3, it can be observed that for t_db_ values higher than the TNZ (25 °C), the rabbits showed higher RR and ET values. These changes in physiological parameters arise because rabbits are highly susceptible to heat stress, as they have few functional sweat glands and have difficulty in eliminating excess body heat when the environmental temperature is high [7]. During the experimental period, the RR of rabbits submitted to t_db_ values higher than 26.75 °C varied from 72 to 113 mov.min^−1^, and the ET ranged from 28.0 to 29.2 °C. In thermoneutral conditions, a rabbit’s RR can range from 44 to 98 mov.min^−1^ for male and female rabbits, and the ET can range from 26.4 to 26.9 °C [34]. When t_db_ is above the TNZ, a number of adverse impacts on the animal can result, such as reduced energy intake and metabolic rate [28], which, in most cases, are accompanied by reduced performance, such as reduced growth rate, elevated mortality, and reduced resistance to disease [35]. Furthermore, according to [36], high t_db_ values can cause drastic changes in the reproductive performance of female rabbits, such as a reduced conception rate, altered embryonic development, reduced milk production, changes in age at puberty, and increased pre- and post-weaning mortality. In males, high t_db_ values can cause decreased testosterone concentrations, reductions in ejaculate volume, concentration, and quality; and changes in sexual desire. Figure 2 shows that when t_db_ was lower than 26.75 °C, the RR was lower (from 68 to 86 mov.min^−1^); moreover, Figure 3 shows that when t_db_ was lower than 25.75 °C, the ET ranged from 20.3 to 26.2 °C. Based on the physical responses (ET and RR), these results indicate that the rabbits are in a thermal comfort situation.

When t_db_ is below the TNZ, a negative influence is exerted on the animals. Similar to findings for other animals, in cold challenge conditions, part of the animal’s feed energy intake that could be used for growth or production is diverted to thermoregulation to maintain homeostasis [37]. For a severely low t_db_, animals can experience hypothermia, decreased alertness, and behavioural and physiological disorders [38].

In addition to t_db_, the RH is an essential parameter indicating thermal conditions for rabbits. Rabbits are sensitive to very low humidity (below 55%) but not to very high humidity [31]. During most of the experimental period, the RH was high, and the humidity level was not found to adversely affect the rabbits at moderate temperatures. However, when t_db_ is too high (close to the rabbit’s body temperature), a high RH can be a problem. According to [10], in this situation, latent heat dissipation becomes inefficient because it is dependent on the RH. The dissipation of heat through respiratory water vapour is decreased by the increase in ambient RH, resulting in discomfort and potential prostration [31]. Unfortunately, this is a common situation in tropical climates during the rainy season.

According to Figure 3, the decision tree can be interpreted as reported in Table 5.

In addition to these descriptive statistical indices, functional relationships between the RR and ET values predicted by the decision trees and the values observed during the experiment period were compared, resulting in the respective equations (Equations (1) and (2)).

(1) $$RR_{simulated\;by\;DT}=0.90\times RR_{Observed}+8.09\;\left(Standard\;error=\pm 2.01\right)$$

(2) $$ET_{simulated\;by\;DT}=1.014\times ET_{Observed}-0.38\;\left(Standard\;error=\pm 0.12\right)$$

**Figure 4:**
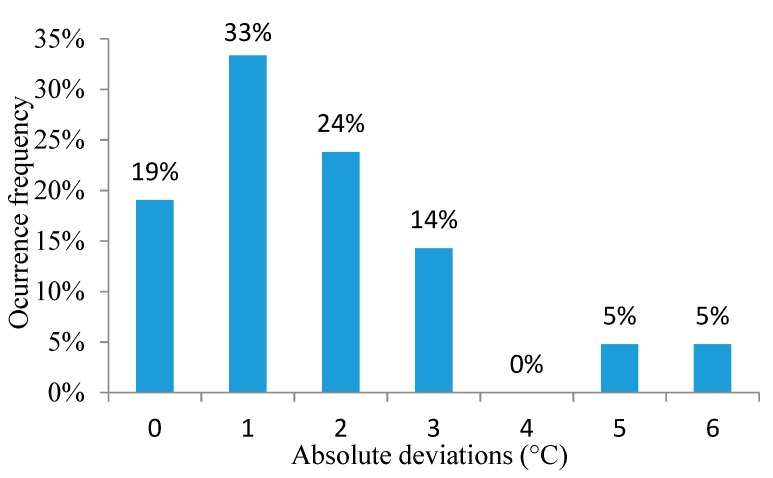
Occurrence frequencies for absolute deviation of the RR.

**Figure 5:**
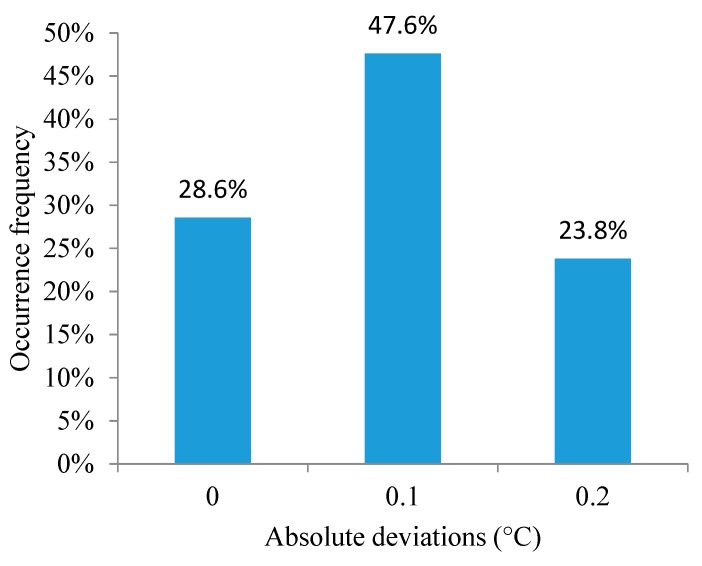
Occurrence frequencies for absolute deviation of the ET.

According to [39], if the intercept is close to 0 and the slope is close to 1, then the accuracy is relatively high. The slope value in Equation (1) is close to 1, while the slope value for Equation (2) is equal to 1, but the intercept value in Equation (1) is not close to 0. In Equation (2), the intercept value is close to 0, indicating a better precision in the prediction of the output values [39]. Figure 4 and Figure 5 show histograms of the occurrence frequencies for the absolute deviation.

From 60 decision tree models, two final models were selected to predict two physiological output variables: the RR and ET. The results show that the predicted values from the two final models are very similar to the experimentally observed values. The ET model exhibited better statistical indices than the RR model. The statistical indices indicate that the prediction errors of the decision tree for RR are concentrated (76%) over a smaller range of absolute deviation, from 0 to 2 mov.min^−1^. The remaining 24% of the absolute deviations were concentrated from 3 to 6 mov.min^−1^. For the ET model, 76.3% of the prediction errors were concentrated in an absolute deviation range of 0–0.1 °C. The remaining 23.8% of the absolute deviations are concentrated at 0.2 °C. Together, these results indicate an excellent predictive capacity of the decision tree models.

The decision trees developed in this research can be used in practical situations to provide a rapid evaluation of rabbit welfare conditions. The decision trees enable one to estimate the physiological responses (ET and RR) of rabbits under different thermal conditions based on t_db_ and RH. This information can be obtained in real time and may help in decision-making to maintain satisfactory environmental conditions for rabbits. Therefore, these physical responses may act as indicators of thermal comfort or discomfort of the animals. If the rabbits are determined to be in discomfort conditions based on their physical responses, it can be inferred that this thermal stress situation may cause an imbalance in their body temperature and hence adversely affect growth and reproduction [7,36]. In addition, animals reared under thermal stress can suffer losses in performance and production due to eating disorders, weight gain, feed conversion, water consumption, changes in blood parameters and enzymatic and hormonal reactions in addition to the imbalance of proteins, energy, and minerals [40,41]. Therefore, the proposed decision trees can be used to evaluate the physiological responses of rabbits to their thermal environment in order to better understand this relationship, leading to an enhancement of both animal welfare and production efficiency [1]. In this sense, for applications in intensive rabbit breeding, in an attempt to mitigate the harmful effects of environmental stress, the housing characteristics and equipment must be adequate to provide appropriate comfort to the animals, while also supporting the management of breeding, providing greater financial gain for the producer [1].

## 4. Conclusions

Decision tree models were successfully employed to predict physiological variables, based on ranges of input variable values and decision-making rules determined from the data.

Environmental variables (such as t_db_ and RH) and physiologic variables (such as RR and ET) can be evaluated as a knowledge-based management subsystem in a decision support system, which provides information about data and potential relationships to assist in decision-making.

Additionally, it was shown that the decision trees can assist in predicting physiological responses. The model exhibited a good fit between the observed and predicted data. Thus, the final decision trees can support decision-making to avoid thermal stress conditions.

## Figures and Tables

**Table 1:** Mean experimental values for validation of the decision tree models.

Input Variables		Output Variables			
t_db_ (°C)	RH (%)	RR (mov.min^−1^)	SD	ET (°C)	SD
23.4	76	83	±14.1	23.8	±1.8
23.5	70	71	±15.8	22.9	±2.1
23.6	77	78	±16.5	24.2	±1.5
23.6	74	76	±14.3	20.2	±1.5
23.8	86	72	±20.7	24.2	±1.6
24.5	78	75	±11.9	25.8	±1.5
24.5	87	81	±22.1	24.2	±1.9
24.7	77	70	±18.6	24.6	±2.0
25.0	73	86	±17.4	25.4	±1.9
25.4	84	81	±21.8	26.4	±1.7
25.5	80	86	±14.5	24.7	±1.7
26.0	73	82	±22.5	28.1	±1.0
27.5	72	107	±23.3	28.5	±0.9
27.6	81	108	±28.1	28.8	±0.9
27.7	73	97	±28.2	29.3	±1.0
27.7	74	72	±12.7	28.1	±1.2
27.8	84	88	±23.4	28.8	±1.5
27.9	82	83	±32.6	29.2	±0.9
28.0	75	109	±23.2	28.9	±1.0
28.1	77	98	±23.4	28.7	±1.3
29.2	67	99	±28.4	29.1	±1.2

t_db_: dry bulb temperature. RH: relative humidity. RR: respiratory rate. ET: ear temperature. SD: standard deviation.

**Table 2:** Decision tree parameters for ET.

Parameter	Value
SplitCriterion	‘mse’
MinParent	10
MinLeaf	5
MaxSplits	381
NVarToSample	‘all’
MergeLeaves	‘on’
Prune	‘on’
PruneCriterion	‘mse’
QEToler	1.00 × 10^−6^
NSurrogate	0
MaxCat	10
AlgCat	‘auto’
PredictorSelection	‘interaction-curvature’
UseChisqTest	1
Stream	[]
Version	2
Method	‘tree’
Type	‘regression’

mse: mean square error.

**Table 3:** RR decision tree parameters.

Parameter	Value
SplitCriterion	‘mse’
MinParent	14
MinLeaf	7
MaxSplits	381
NVarToSample	‘all’
MergeLeaves	‘on’
Prune	‘on’
PruneCriterion	‘mse’
QEToler	1.00 × 10^−6^
NSurrogate	0
MaxCat	10
AlgCat	‘auto’
PredictorSelection	‘allsplits’
UseChisqTest	1
Stream	[]
Version	2
Method	‘tree’
Type	‘regression’

mse: mean square error.

**Table 4:** Statistical indices for test results for the decision tree models.

Input Variables		Output Variables									
t_db_ (°C)	RH (%)	Observed RR (mov.m^−1^)	Predicted RR (mov.m^−1^)	Absolute Deviation	Standard Deviation	Percentage Error	Observed ET (°C)	Predicted ET (°C)	Absolute Deviation	Standard Deviation	Percentage Error
23.4	76	83	83	0	0.00	0.00	23.8	23.6	0.2	0.14	0.84
23.5	70	71	72	1	0.71	1.41	22.9	23.1	0.2	0.14	0.87
23.6	77	78	73	5	3.54	6.41	24.2	24.3	0.1	0.07	0.41
23.6	74	76	74	2	1.41	2.63	20.2	20.3	0.1	0.07	0.50
23.8	86	72	71	1	0.71	1.39	24.2	24.3	0.1	0.07	0.41
24.5	78	75	76	1	0.71	1.33	25.8	25.9	0.1	0.07	0.39
24.5	87	81	80	1	0.71	1.23	24.2	24.3	0.1	0.07	0.41
24.7	77	70	70	0	0.00	0.00	24.6	24.6	0	0.00	0.00
25.0	73	86	89	3	2.12	3.49	25.4	25.6	0.2	0.14	0.79
25.4	84	81	83	2	1.41	2.47	26.4	26.2	0.2	0.14	0.76
25.5	80	86	85	1	0.71	1.16	24.7	24.6	0.1	0.07	0.40
26.0	73	82	80	2	1.41	2.44	28.1	28.2	0.1	0.07	0.36
27.5	72	107	113	6	4.24	5.61	28.5	28.4	0.1	0.07	0.35
27.6	81	108	108	0	0.00	0.00	28.8	28.8	0	0.00	0.00
27.7	73	97	98	1	0.71	1.03	29.3	29.2	0.1	0.07	0.34
27.7	74	72	72	0	0.00	0.00	28.1	28.1	0	0.00	0.00
27.8	84	88	91	3	2.12	3.41	28.8	28.9	0.1	0.07	0.35
27.9	82	83	82	1	0.71	1.20	29.2	29.2	0	0.00	0.00
28.0	75	109	111	2	1.41	1.83	28.9	28.7	0.2	0.14	0.69
28.1	77	98	101	3	2.12	3.06	28.7	28.7	0	0.00	0.00
29.2	67	99	97	2	1.41	2.02	29.1	29.1	0	0.00	0.00
Mean:				1.8	1.2	2.0			0.1	0.1	0.4
Minimum:				0	0	0			0	0	0
Median:				1	0.71	1.41			0.1	0.07	0.39
Maximum:				6	4.24	6.41			0.2	0.14	0.87
Standard error:				2.01					0.1		
RMSE:				2.34					0.1		
R^2^:				97.5%					99.8%		

t_db_: dry bulb temperature. RH: relative humidity. RR: respiratory rate. ET: ear temperature.

**Table 5:** Decision-making rule base.

Node	Antecedent		Then Part	Consequence		Antecedent		Then Part	Consequence			
1	if	t_db_ < 25.75	then	node	2	elseif	t_db_ ≥ 25.75	then	node	3	else	26.4
2	if	t_db_ < 23.7	then	node	4	elseif	t_db_ ≥ 23.7	then	node	5	else	24.3
3	if	t_db_ < 27.55	then	node	6	elseif	t_db_ ≥ 27.55	then	node	7	else	28.7
4	if	RH < 75	then	node	8	elseif	RH ≥ 75	then	node	9	else	22.9
5	if	RH < 85	then	node	10	elseif	RH ≥ 85	then	node	11	else	25.1
6	if	t_db_ < 26.75	then	node	12	elseif	t_db_ ≥ 26.75	then	node	13	else	28.3
7	if	RH < 73.5	then	node	14	elseif	RH ≥ 73.5	then	node	15	else	28.8
8	if	t_db_ < 23.55	then	node	16	elseif	t_db_ ≥ 23.55	then	node	17	else	21.8
9	if	t_db_ < 23.5	then	node	18	elseif	t_db_ ≥ 23.5	then	node	19	else	23.9
10	if	RH < 82	then	node	20	elseif	RH ≥ 82	then	node	21	else	25.4
11	fit	=	24.3									
12	fit	=	28.2									
13	fit	=	28.4									
14	if	t_db_ < 28.45	then	node	22	elseif	t_db_ ≥ 28.45	then	node	23	else	29.2
15	if	RH < 74.5	then	node	24	elseif	RH ≥ 74.5	then	node	25	else	28.7
16	fit	=	23.1									
17	fit	=	20.3									
18	fit	=	23.6									
19	fit	=	24.3									
20	if	t_db_ < 24.6	then	node	26	elseif	t_db_ ≥ 24.6	then	node	27	else	25.2
21	fit	=	26.2									
22	fit	=	29.2									
23	fit	=	29.1									
24	fit	=	28.1									
25	if	t_db_ < 27.95	then	node	28	elseif	t_db_ ≥ 27.95	then	node	29	else	28.8
26	fit	=	25.9									
27	if	RH < 75	then	node	30	elseif	RH ≥ 75	then	node	31	else	24.9
28	if	t_db_ < 27.85	then	node	32	elseif	t_db_ ≥ 27.85	then	node	33	else	28.9
29	fit	=	28.7									
30	fit	=	25.6									
31	fit	=	24.6									
32	if	t_db_ < 27.7	then	node	34	elseif	t_db_ ≥ 27.7	then	node	35	else	28.8
33	fit	=	29.2									
34	fit	=	28.8									
35	fit	=	28.9									

t_db_: dry bulb temperature. RH: relative humidity.
